# The State in Relation to Chronic Disease

**Published:** 1919-12-27

**Authors:** 


### THE STATE IN RELATION TO CHRONIC DISEASE.

In his Chadwick Public Lecture, delivered on Thursday, November 13, at 5.15 p.m., at the rooms of the Medical Society of London, Chandos Street, W., Dr. R. O. Moon explained that the first duty of the State in connection with disease is prevention. This duty was well recognised by the Royal Army Medical Corps in the war, though many activities were hampered by inelastic regulations. Primary duties with regard to prevention are:

(*a*) To secure a pure water supply.
(*b*) To supply unadulterated food and good milk.
(*c*) Removal of dust and smoke.
(*d*) House and town planning.
(*e*) Restriction of the consumption of alcohol.

The lecturer quoted the low rate of infant mortality at Letchworth as an example of the benefit of town-planning. Proceeding, Dr. Moon said the chronic diseases at present dealt with by the State are insanity and tuberculosis. There was a great need for hospitals or special houses for borderland cases. The exaggerated idea of the infectivity of "consumption" which possesses the public mind has to be combated. Post-sanatorium treatment for the tuberculous should be amply provided. Dr. Moon dwelt on the importance of civic efficiency to the nation, and pointed out ways of dealing with inefficiency resulting from circulatory disorders and epilepsy. Similar work to that which is being done by employment bureaux for disabled soldiers should be applied to all citizens suffering from chronic disease. Doctors recommend "light work," but there is difficulty in defining it, and medical men should become familiar with the amount of work involved in various forms of industrial and agricultural occupations.

In conclusion, the lecturer pleaded for closer co-operation between Pensions Committees, Labour Exchanges, and the medical profession. After the lecture Sir James Crichton-Browne presented the Chadwick Gold Medal and Prize for Excellence in Municipal Engineering and Hygiene to Mr. P. S. J. Bovey, a matriculated student and prizewinner in the Engineering Section at University College.

The two remaining lectures of the Chadwick autumn programme for this year are "Some Problems of Suburban Housing," by Captain R. Reiss, F.R.Econ.S., at the Central Library, Finchley Road, Hampstead, on Monday, the 17th inst., at 8 p.m.; and "Causes of Infant Mortality," by Dr. Joseph Cates, of St. Helens, at the Hampstead Town Hall, Haverstock Hill, on Thursday, December 4, at 8 p.m. Admission is free to all Chadwick Lectures.

